# RNA-seq Characterization of Melanoma Phenotype Switch in 3D Collagen after p38 MAPK Inhibitor Treatment

**DOI:** 10.3390/biom11030449

**Published:** 2021-03-17

**Authors:** Vladimír Čermák, Aneta Škarková, Ladislav Merta, Veronika Kolomazníková, Veronika Palušová, Stjepan Uldrijan, Daniel Rösel, Jan Brábek

**Affiliations:** 1 Department of Cell Biology, Charles University, Viničná 7, 128 44 Prague, Czech Republic; vladimir.cermak@natur.cuni.cz (V.Č.); aneta.skarkova@natur.cuni.cz (A.Š.); ladislav.merta@natur.cuni.cz (L.M.); veronika.kolomaznikova@natur.cuni.cz (V.K.); daniel.rosel@natur.cuni.cz (D.R.); 2 Biotechnology and Biomedicine Centre of the Academy of Sciences and Charles University (BIOCEV), Průmyslová 595, 252 42 Vestec u Prahy, Czech Republic; 3 Department of Biology, Faculty of Medicine, Masaryk University, Kamenice 5, 625 00 Brno, Czech Republic; ver.palusova@mail.muni.cz (V.P.); uldrijan@med.muni.cz (S.U.); 4 International Clinical Research Center, St. Anne’s University Hospital, Pekařská 53, 656 91 Brno, Czech Republic

**Keywords:** cancer, melanoma, metastasis, phenotype switch, amoeboid invasion

## Abstract

Melanoma phenotype plasticity underlies tumour dissemination and resistance to therapy, yet its regulation is incompletely understood. In vivo switching between a more differentiated, proliferative phenotype and a dedifferentiated, invasive phenotype is directed by the tumour microenvironment. We found that treatment of partially dedifferentiated, invasive A375M2 cells with two structurally unrelated p38 MAPK inhibitors, SB2021920 and BIRB796, induces a phenotype switch in 3D collagen, as documented by increased expression of melanocyte differentiation markers and a loss of invasive phenotype markers. The phenotype is accompanied by morphological change corresponding to amoeboid–mesenchymal transition. We performed RNA sequencing with an Illumina HiSeq platform to fully characterise transcriptome changes underlying the switch. Gene expression results obtained with RNA-seq were validated by comparing them with RT-qPCR. Transcriptomic data generated in the study will extend the present understanding of phenotype plasticity in melanoma and its contribution to invasion and metastasis.

## 1. Introduction

Despite significant progress in therapy, melanoma remains a life-threatening disease with a high risk of early metastasis. Understanding the biological processes underlying melanoma metastasis is expected to generate new advances and therapeutic modalities in melanoma management [1]. Melanoma is a cancer arising from melanocytes, pigment-producing cells located in the epidermis and a few other tissues. It shows unique biological features related to its developmental origin, namely specific differentiation plasticity and migratory behaviour. The central or master regulator of the melanocyte lineage is the transcription factor MITF [2,3]. MITF regulates expression of pigmentation-related genes, and coordinates differentiation and proliferation in developing melanocytes. In addition, it affects many other biological processes, including cell survival, invasion, senescence, metabolism, autophagy, lysosomal biogenesis, and DNA damage repair [2]. MITF is regulated at transcription and multiple post-transcriptional levels. Its transcription in the melanocyte lineage is driven by SOX10, WNT/β-catenin and CREB, and is directly or indirectly suppressed by NF-κB, JNK, hedgehog, TGFβ, activated RhoA, HIF1α, Brn2/POU3F2 and ATF4 [2,3,4,5,6]. 

**Figure 1:**
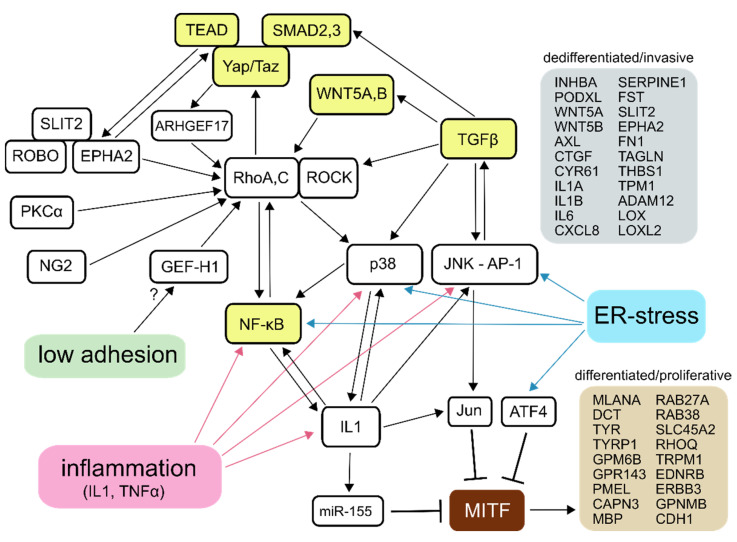
Signalling circuitry of the invasive melanoma phenotype. Essential, broadly accepted regulators are marked by yellow colour. Other regulators, relationships and responses to stimuli were compiled from the literature related to both invasive–proliferative phenotype plasticity and amoeboid invasion. Listed examples of genes whose high expression is characteristic of either phenotype were assembled from the published literature and datasets.

A large-scale comparative study of published microarray data from melanoma cell lines identified two distinct gene expression profiles and associated phenotypes that were termed “invasive” and “proliferative” [7]. The different gene expression patterns correspond to low and high MITF activity and the related differentiation status of the cells. The dedifferentiated, invasive phenotype was originally characterised by a sustained activation of TGFβ target genes, a shift from canonical to non-canonical WNT signalling, and a low expression of MITF-regulated genes. Later, it was found that it also involves sustained NF-κB, Yap/Taz-TEAD, ER-stress and Rho-ROCK signalling [5,7,8,9,10,11]. Melanoma dedifferentiation and invasive phenotype can be induced or enhanced by inflammatory factors (IL1, TNFα), TGFβ, glutamine starvation, conditions of low adhesion (e.g., growth in melanospheres) and hypoxia [5,12,13,14,15,16,17,18]. It is associated with resistance to both BRAF inhibitors and immunotherapy, as well as with enhanced metastasis and poor prognosis [10,17,18,19,20,21]. The underlying signalling pathways, their cross-talks and feed-forward loops responsible for, or, presumably, contributing to, the preservation of the invasive phenotype in melanoma cells and examples of transcripts specifically associated with either phenotype, are summarised in Figure 1 [8,11,22,23,24,25,26,27,28,29,30,31,32,33,34,35].

Cancer cells, including melanoma, can individually invade a 3D environment in either a mesenchymal mode, which depends on integrin-mediated adhesion and extracellular protease activity, or in an amoeboid mode that is independent of integrins and localised proteolysis, and is also characterised by largely dissociated microtubules and high RhoA-ROCK-dependent contractility [36,37,38]. As RhoA-ROCK signalling is also required for mesenchymal invasion, it is an attractive target for antimetastatic therapy [39,40]. A375M2 metastatic melanoma cells have been a popular model for studying amoeboid invasion and its regulation, for they show prominent blebbing amoeboid morphology in, or on top of, soft collagen matrices, good long-term viability under such conditions, and, importantly, display substantial plasticity in terms of induced migratory phenotype transitions between amoeboid and mesenchymal modes, referred to as invasion plasticity. So far, amoeboid–mesenchymal transition (AMT) in A375M2 has been achieved by chemical inhibition of ROCK kinases, or gene targeting or knockdowns of miscellaneous regulators involved in the underlying signalling network [29,30,41,42,43,44,45]. Here, we show, for the first time, that treatment of A375M2 cells embedded in a 3D collagen matrix with compounds SB202190 and BIRB796 induces a phenotype switch, as documented by decreased expression of dedifferentiated/invasive phenotype-associated genes and upregulation of differentiated/proliferative phenotype-associated genes. The differentiation phenotype switch is accompanied by a morphological shift from a rounded to an elongated shape, which is indicative of AMT, suggesting that melanoma phenotype plasticity might be interconnected with invasion plasticity. SB202190 is more potent than BIRB796 in all observed effects, particularly in the induction of differentiation-related markers. While both compounds have been developed and used as p38 MAPK inhibitors, they can significantly inhibit several p38-unrelated kinases (see Table 1) [46,47]. Some of these kinases may affect melanoma cell phenotype, as exemplified by SB202190-specific inhibition of BRAF [48]. Recently, an important property distinguishing SB202190 from BIRB796 was identified: a SB202190-specific, p38-independent ability to robustly translocate lysosomal membrane-bound TFEB, a MITF-related protein, to the nucleus of melanoma cells [48]. The same effect was observed for TFEB and TFE3 (another MITF family member) in non-melanoma cells [49]. We confirmed that SB202190, but not BIRB796, elicits nuclear translocation of the MITF-A isoform in melanoma cells (see Figshare file, “Subcellular localisation of MITF” [50]). While MITF-M, the major isoform in melanoma/melanocytes, is constitutively localised in the nucleus, the localisation is not regulated by mTORC1, unlike the other MITF isoforms and TFEB, and does not change after SB202190 or BIRB796 treatment; it forms heterodimers with the other isoforms and with TFEB, TFEC and TFE3 [51,52]. Massive nuclear translocation of these proteins due to SB202190 treatment thus might potentiate transactivation by MITF-M or replace it in the transactivating complexes. Indeed, MITF-A was found to be a more potent activator of DCT than MITF-M, and TFE3 may replace MITF-M in the complex with LEF-1 to activate the DCT promoter [53,54].

## 2. Materials and Methods

### 2.1. Cells Culture, Treatments and Morphological Analysis

A375M2 cells were maintained in DMEM (4.5 g/L glucose, pyruvate) supplemented with 10% foetal bovine serum and 50 μg/mL gentamicin (all from Sigma-Aldrich, St. Louis, MO, USA) at 37 °C in a humidified atmosphere with 5% CO_2_. The cultures were regularly tested for mycoplasma contamination. 3D cell culture experiments were performed with rat tail collagen (SERVA, Heidelberg, Germany) at a concentration of 1 mg/mL and DMEM supplemented with 1% foetal bovine serum, 15mM HEPES and 50 μg/mL gentamicin. In all experiments, cells were treated with 10 μM SB202190 (Santa Cruz Biotechnology, Santa Cruz, CA, USA), 10 μM BIRB796 alias doramapimod (LC Laboratories), or an equivalent volume of DMSO only (0.1% final concentration). To quantify cell morphology in the 3D collagen matrix, 100,000 cells were seeded in 250 μL of collagen in a 48-well plate. Hoffman modulation contrast microscopy images were taken 48 h later from approx. 20 planes along the z-axis. Cells were classified as elongated or rounded as described previously [55]. Please note that different A375-derived cell lines with almost identical names (A375M2, A375 M-2, A375M, etc.) were created in similar ways in a few completely independent studies [56,57]. A375M2 cells used in this study are those from Clark et al., and were kindly provided by Prof. Richard Hynes.

### 2.2. RNA Extraction and Sequencing

A total of 330,000 A375M2 cells were cultured in 500 μL of 3D collagen gel for 48 h in a 24-well plate. RNA was isolated as described in detail previously [55]. Polyadenylated RNA was enriched with Oligo d(T)_25_ Magnetic Beads (New England Biolabs, Ipswich, MA, USA), according to the manufacturer’s instructions. A stranded, Illumina HiSeq-compatible library was constructed with a ScriptSeq Complete (Human/Mouse/Rat) library preparation kit (Epicentre), according to the manufacturer’s instructions. The quality and size distribution of sequencing libraries was verified with P5-P7 PCR, and the concentrations were determined using a PicoGreen (Thermo Fisher Scientific, Waltham, MA, USA) fluorescence measurement. An equimolar pool of 12 sample libraries was sequenced on one whole lane of an Illumina HiSeq 2000/2500 series sequencer in high output, paired mode (2 × 125 cycles). Raw reads were trimmed from adapter sequences with Cutadapt [58] (version 1.15), quality checked with FastQC, and mapped to human genome version GRCh38.100 with the STAR short read aligner [59] version 2.7.4a, with default settings and output extended with read counts per gene. Complete adapter-trimmed fastq data are available from the ArrayExpress database at EMBL-EBI under accession number E-MTAB-9273 [60].

### 2.3. Reverse Transcription–Quantitative Polymerase Chain Reaction (RT-qPCR)

All the RT-qPCR experiments were performed according to MIQE guidelines [61]. Briefly, total RNA was extracted from cells embedded in 3D collagen matrix as described above. RNA reverse transcription was performed using M-MuLV Reverse Transcriptase (NEB) with 1 µM oligo(dT) and (dN)_15_ primer and 1.5 µg of total RNA. SYBR green-based qPCR was performed in a LightCycler 480 Instrument (Roche). For primer details, see the file, PCR.primers.xlsx, available from Figshare [50]. Cq and relative expression values were calculated in R with ReadqPCR and NormqPCR packages, using TARDBP and THRAP3 as reference gene indices (selected based on geNORM analysis) [62,63]. Significance of differences was analysed with two-way ANOVA and pairwise paired *t*-tests, with *p*-value adjustment for multiple testing (Benjamini–Hochberg [64]).

### 2.4. Statistical Analysis

To estimate differential gene expression from RNA sequencing data, a workflow based on the STAR aligner and DESeq2 R package was used, as described previously [65]. We used rlog-transformed values for principal component analysis. Gene set enrichment analysis was performed with Enrichr [66]. We utilised core R functions to compare data produced in this work against two public datasets (Verfaillie et al. 2015 and Folberg et al. 2006) [11,67]. We calculated the numbers of shared genes (represented by gene symbols) for all possible pairs of gene sets, and the related Jaccard indices and hypergeometric right-tail cumulative *p*-values, to estimate the statistical significance of the intersections. As an approximation, we used the total number of all HGNC gene symbols assigned to protein-coding and non-coding RNA genes (27,371) as the size of the source population in hypergeometric tests. Venn diagrams representing intersections of the gene sets were generated using a Multiple List Comparator online tool [68].

## 3. Results

### 3.1. SB202190 and BIRB796 Induce a Phenotype Switch in A375M2 Cells Cultured in 3D Collagen

**Figure 2:**
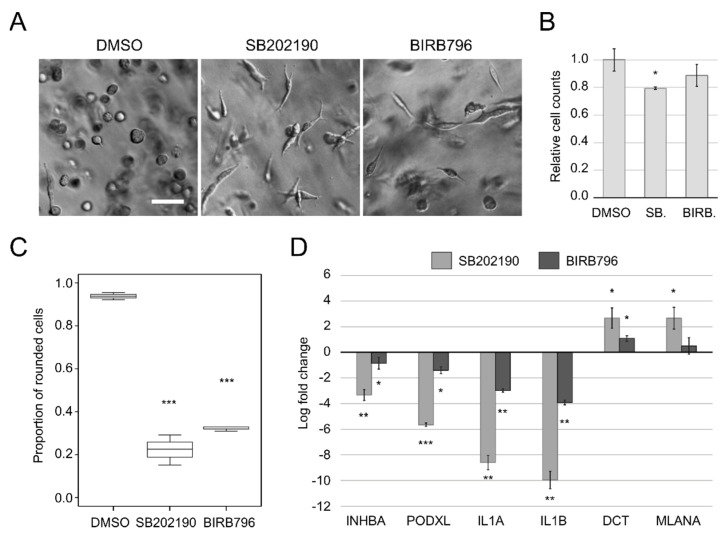
Phenotype switch induced by SB202190 and BIRB796 in A375M2 cultured in 3D collagen. (**A**) Representative wide-field images of cells in 3D collagen treated with DMSO, SB202190 or BIRB796. Scale bar: 75 µm. (**B**) Numbers of live cells after 48 h of the indicated treatments counted in equal volumes of 3D gels and normalised to DMSO controls (SB—SB202190, BIRB—BIRB796). Averages of three independent biological replicates; error bars: standard deviation. (**C**) Quantification of cell morphology in 3D collagen (three biological replicates, logistic regression with Wald test). (**D**) RT-qPCR detection of changes in expression of the indicated genes. Averages of three independent biological replicates; error bars: standard deviation. *p*-values: *** *p* < 0.001, ** *p* < 0.01, * *p* < 0.05.

Treatment of A375M2 melanoma cells with 10 µM compounds SB202190 and BIRB796 for 48 h in 3D collagen resulted in an elongated shape in the majority of the cell population (Figure 2A,C), also see Figshare item, “Bright field microscopy photographs of A375M2 3D cell cultures”). The compounds were well tolerated, with only a mild decline in cell numbers after SB202190 treatment (Figure 2B). RT-qPCR analysis of transcripts associated with the dedifferentiated/invasive phenotype (INHBA, PODXL, IL1A, IL1B), and of MITF-regulated genes (DCT, MLANA) associated with the differentiated/proliferative phenotype, confirmed a phenotype switch consistent with the characteristic gene expression signature described in the literature (Figure 2D). SB202190 was more potent than BIRB796 in both morphological and gene expression level effects.

### 3.2. Transcriptomic Profiling of the Phenotype Switch-Associated Changes with RNA-seq

**Figure 3:**
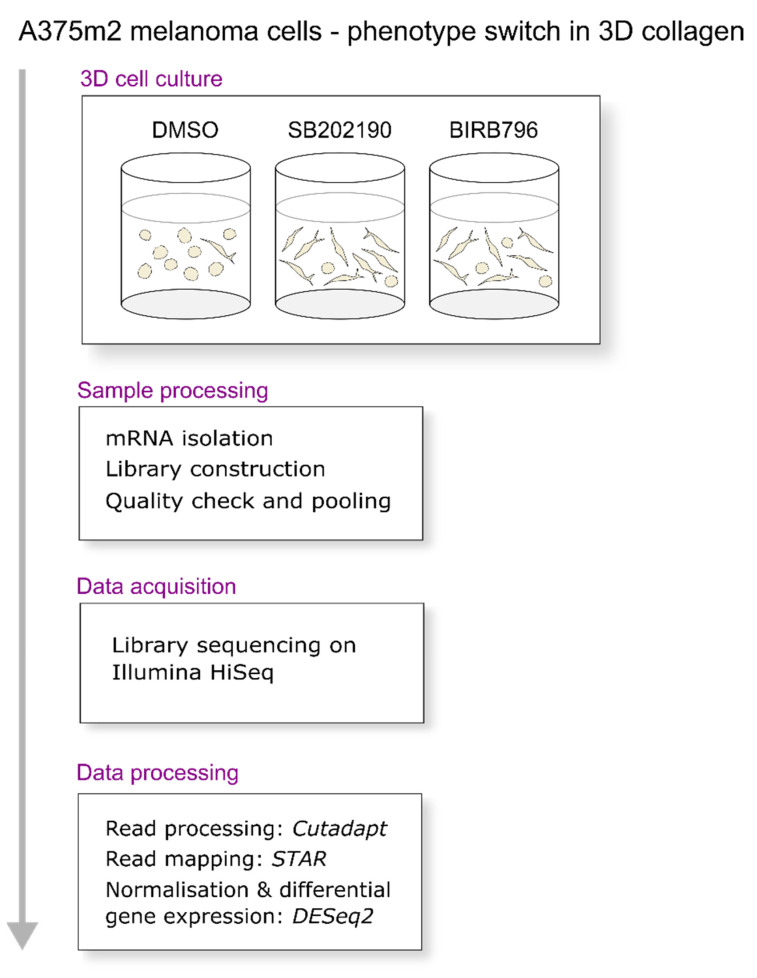
Data acquisition and processing strategy.

To uncover the transcriptomic changes underlying the dual phenotype switch, we performed RNA sequencing of mRNA samples from SB202190, BIRB796 or DMSO-treated cells prepared in four independent biological replicates. The cells were kept for 48 h in 3D collagen with the compounds, and then the whole samples, including the collagen, were homogenised and further processed for RNA sequencing. Polyadenylated RNA was enriched with oligo-dT magnetic beads, converted into a stranded cDNA library, and sequenced with an Illumina HiSeq sequencer (Figure 3).

**Figure 4:**
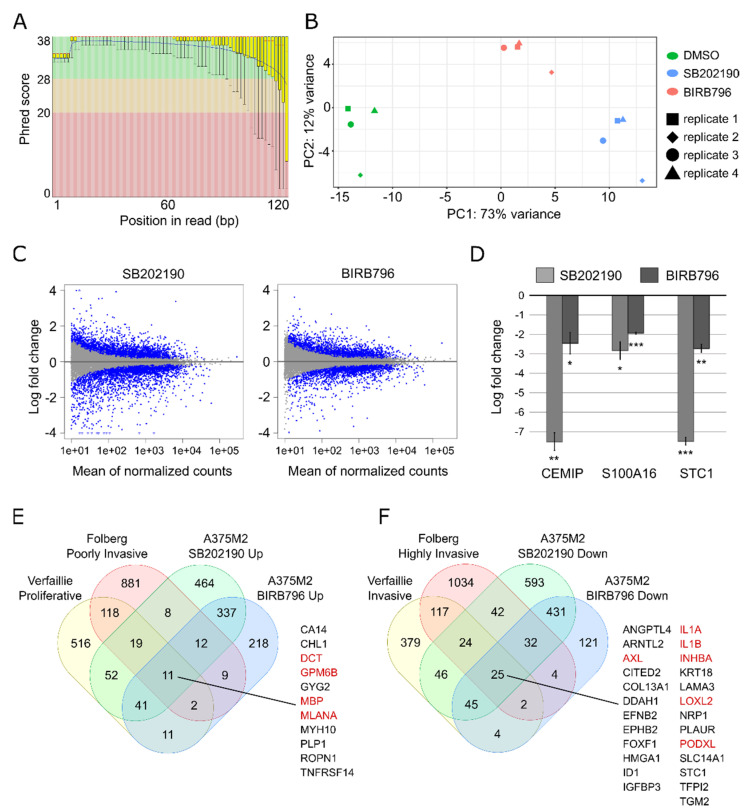
Technical validation of RNA sequencing results. (**A**) Per base sequence quality of RNA sequencing reads expressed as Phred score by position, sample 1, first reads. (**B**) Principal component analysis of gene expression profiles. (**C**) MA plot of log2 fold change values against normalised counts for each gene in the analysis. Blue points mark genes with FDR<0.1. (**D**) Log2 fold change values of the indicated genes detected by RT-qPCR in three samples independent from the RNA-seq series. Error bars: standard deviation. Adjusted *p*-values: *** *p* < 0.001, ** *p* < 0.01, * *p* < 0.05. (**E**,**F**) Venn diagrams representing intersections of gene lists from the indicated datasets. Highlighted in red are the genes whose higher expression is well known to be characteristic of either the differentiated/proliferative (**E**) or of the dedifferentiated/invasive phenotype (**F**).

The sequencing yielded approx. 21–26.5 million paired-end reads per sample. Raw reads trimmed from adapter sequences were quality checked with FastQC software [69]. FastQC’s plots of Phred scores by position showed typical profiles with decreasing quality towards the ends of reads (Figure 4A).

The mapping metrics of the STAR aligner showed an average paired read length of 240 nucleotides. On average, the aligner uniquely mapped 93.6% of the fragments to the human genome; 3.1% of the fragments were multi-mapped and 3.1% of the fragments were excluded for being too short. For the complete mapping metrics, see Figshare file, “RNA sequencing raw data and mapping metrics” [50]. Principal component analysis of normalised gene expression profiles showed that the treatments were the dominant source of variation (Figure 4B). Differential gene expression analysis with DESeq2 [70] identified that 2700 out of 16,612 Ensembl genes (16.3%) changed with adjusted *p*-value < 0.1 and fold change > 1.5 after treatment with SB202190. BIRB796 treatment changed the expression of 1638 out of 16,612 (9.9%) genes. The distribution of affected transcripts by gene expression level is depicted as an MA plot in Figure 4C. Complete results are available from Figshare (“Differential gene expression analysis”) [50]. Selected differentially expressed genes from the gene lists, characteristic of either invasive or proliferative phenotypes (see Figure 1), are listed in Table 2.

Next, we analysed the differentially expressed genes for over-representation of gene lists from published datasets using the Enrichr web server (Table 3). We found that the genes upregulated by SB202190 and BIRB796 are significantly enriched with genes with high expression in a subset of melanoma cell lines from the NCI-60 cancer cell line panel. Importantly, all the cell lines in this subset were previously classified as “proliferative” within a large panel of melanoma cell lines, see the HOPP database [71]. The genes downregulated by the compounds showed many more concordant enrichments; Table 3 displays the top enriched GO Biological Process terms and TRRUST transcriptional regulatory networks [72]. For complete results, see the Figshare file, “Gene set enrichments”.

### 3.3. Validation and Reproducibility of the RNA-seq Results

To validate the biological reproducibility of the results of differential gene expression analysis, we performed RT-qPCR measurements of three selected transcripts—CEMIP, S100A16 and STC1—in RNA samples independent from those used for the RNA sequencing (three biological replicates). The corresponding genes were identified as significantly differentially expressed in both SB202190 and BIRB796 data, and are also present in published gene expression signatures of the invasive phenotype, e.g., S100A16 in Verfaillie et al. 2015, Tirosh et al. 2016 and Jeffs et al. 2009 [11,73,74]. The results confirmed that the genes were similarly affected in the independent samples (Figure 4D). We also wondered to what extent are the data generated in the presented study matching the published gene expression signatures characterising the phenotype switch. For comparison, we chose two published gene expression profiles of invasive and proliferative phenotypes with a total number of affected genes similar to our study—Verfaillie et al. 2015 and Folberg et al. 2006 [11,67]. The comparison revealed that the two published studies overlap, with the data from SB202190-treated A375M2 cells being similar. Data from BIRB796-treated cells overlap by approx. half of the numbers, but still significantly (see Figshare data, “Comparison of A375M2 data sets against public datasets” [50]). We also analysed all intersections of the gene lists in detail and found subsets shared by all the lists (11 genes for “proliferative” and 25 genes for “invasive” sets) containing known markers of the respective phenotypes (Figure 4E,F).

## 4. Discussion

The signalling network controlling amoeboid phenotype in melanoma, as has been previously uncovered in A375M2 cells, is virtually identical to the one responsible for the dedifferentiated/invasive phenotype (i.e., NF-κB, TGFβ and non-canonical WNT signalling) [41,42,75]. The amoeboid phenotype is also known to be suppressed by β-catenin-stimulated MITF expression [76]. EPHA2, which is highly expressed in dedifferentiated/invasive melanoma cells, stimulates RhoA activity and promotes amoeboid migration [77]. It thus seems that amoeboid invasion is a manifestation of the dedifferentiated/invasive phenotype observable under suitable 3D conditions (in terms of cell viability and matrix composition). Our data bring essential additional support for such a hypothesis and encourage further large-scale 3D culture-based or intravital analyses required for definitive confirmation. We suggest that the presented data are of high relevance to melanoma biology and cancer metastasis research fields, as they bridge invasion and phenotype plasticity in melanoma cells.

## Figures and Tables

**Table 1:** Targets and off-targets of SB202190 and BIRB796. Plus signs mark K_d_ nM values from ref. 46: + <1000, ++ <100, +++ <10, ++++ <1. Crosses mark residual kinase activity in the presence of 1 µM inhibitor (from ref. 47, x <50%, xx <10%).

Kinase	SB202190	BIRB796
p38alpha	xx/+++	xx/++++
p38beta	xx/++	x/
p38gamma		x/+
p38delta		x
JNK2	x/+	xx/++
JNK3	x/++	
NLK	x/++	
RIPK2/RIP2	xx/+	
GAK	xx/++	
CK1delta	x/++	
BRAF	+	
GSK3beta	x	
CK1epsilon	+	
Lck	x	
ACVR1B	+	
CIT	+	
CDC42BPG	+	
EGFR	+	
PRKACB	+	
RPS6KA1	+	
RPS6KA6	+	
STK36	+	
DDR1		++
TIE1		++
MAP4K4		+
STK10		+
SLK		+
ABL1		+
DDR2		+
TIE2		+
RSK1		x
RSK2		x
BRSK2		x

**Table 2:** Changes in expression of melanoma phenotype-related genes detected with RNA-seq. Average log2 fold change values ± standard error as obtained with DESeq2.

Gene	SB202190	BIRB796
TRPM1	4.00 ± 0.67	
DCT	3.38 ± 0.09	1.37 ± 0.09
MLANA	2.47 ± 0.18	0.94 ± 0.19
GPM6B	2.07 ± 0.08	1.00 ± 0.08
PMEL	1.58 ± 0.27	
TYR	1.39 ± 0.08	
MBP	1.17 ± 0.34	1.52 ± 0.34
RAB27A	0.95 ± 0.11	
CAPN3	0.88 ± 0.13	
GPNMB	0.80 ± 0.07	
IL1B	−4.48 ± 0.33	−3.06 ± 0.29
IL1A	−3.80 ± 0.18	−1.95 ± 0.15
CXCL8	−3.60 ± 0.18	−2.01 ± 0.15
SERPINE1	−3.07 ± 0.58	
PODXL	−2.83 ± 0.16	−1.36 ± 0.15
AXL	−2.70 ± 0.41	−1.11 ± 0.34
INHBA	−2.24 ± 0.12	−0.88 ± 0.12
FN1	−1.91 ± 0.09	−1.23 ± 0.09
LOXL2	−1.81 ± 0.10	−0.78 ± 0.10
FST	−1.61 ± 0.11	−1.13 ± 0.11
ADAM12	−1.34 ± 0.10	−0.60 ± 0.10
WNT5B	−0.82 ± 0.35	
WNT5A	−0.80 ± 0.11	
THBS1	−0.73 ± 0.11	

**Table 3:** Gene set enrichment analysis. Over-representation of gene lists from published datasets, in the differentially expressed genes detected in this study, was analysed using the Enrichr web server. Adjusted *p*-values.

Database	Data	SB202190	BIRB796
NCI-60 cancer cell line panel vs. upregulated genes	UACC257	1.83 × 10^−10^	1.33 × 10^−4^
	SKMEL5	1.83 × 10^−10^	7.06 × 10^−2^
	SKMEL28	1.46 × 10^−6^	2.49 × 10^−3^
	MALME 3M	4.46 × 10^−5^	1.85 × 10^−2^
	M14	5.64 × 10^−3^	1.07 × 10^−1^
GO—Biological Process vs. downregulated genes	extracellular matrix organization (GO:0030198)	3.44 × 10^−20^	2.27 × 10^−11^
	regulation of cell proliferation (GO:0042127)	2.79 × 10^−11^	2.09 × 10^−5^
	regulation of apoptotic process (GO:0042981)	4.35 × 10^−11^	1.38 × 10^−9^
	regulation of cell migration (GO:0030334)	6.14 × 10^−11^	4.86 × 10^−7^
	regulation of angiogenesis (GO:0045765)	8.39 × 10^−9^	3.34 × 10^−5^
	negative regulation of programmed cell death (GO:0043069)	9.99 × 10^−9^	3.34 × 10^−5^
	cellular response to cytokine stimulus (GO:0071345)	1.86 × 10^−8^	2.11 × 10^−4^
	positive regulation of angiogenesis (GO:0045766)	3.98 × 10^−8^	6.02 × 10^−5^
	regulation of signal transduction (GO:0009966)	3.98 × 10^−8^	6.02 × 10^−5^
	negative regulation of apoptotic process (GO:0043066)	7.51 × 10^−8^	2.06 × 10^−5^
	positive regulation of cell migration (GO:0030335)	7.51 × 10^−8^	7.08 × 10^−5^
	regulation of MAPK cascade (GO:0043408)	2.39 × 10^−7^	6.17 × 10^−5^
TRRUST vs. downregulated genes	NFKB1 human	8.76 × 10^−9^	1.85 × 10^−6^
	RELA human	3.55 × 10^−8^	1.08 × 10^−5^
	NFKB1 mouse	7.76 × 10^−8^	1.01 × 10^−11^
	VHL human	5.70 × 10^−7^	5.01 × 10^−4^
	STAT3 mouse	7.85 × 10^−7^	1.08 × 10^−5^
	SP1 mouse	8.33 × 10^−^^7^	1.07 × 10^−11^
	EGR1 mouse	2.54 × 10^−6^	3.75 × 10^−6^
	ETS1 human	2.72 × 10^−6^	4.92 × 10^−5^

## Data Availability

Supporting data referenced in the text, complete results of differential gene expression analysis, and published data comparisons have been deposited in the Figshare repository [50]. Adapter-trimmed RNA sequencing data have been deposited in the ArrayExpress database at EMBL-EBI (accession number E-MTAB-9273) [60]. The scripts used in data processing are available from Figshare [50] (see “Code used in data processing”).
